# Immunoglobulin replacement therapy in patients with primary and secondary immunodeficiencies: impact of infusion method on immunoglobulin-specific perceptions of quality of life and treatment satisfaction

**DOI:** 10.1186/s13223-024-00939-y

**Published:** 2025-01-07

**Authors:** Rajiv Mallick, Noemi Hahn, Christopher Scalchunes

**Affiliations:** 1 CSL Behring, King of Prussia, PA USA; 2 Bryter Inc, New York, NY USA; 3 Immune Deficiency Foundation, Towson, MD USA

**Keywords:** Immunoglobulin replacement therapy, Primary immunodeficiency, Secondary immunodeficiency, Life Quality Index, Immunoglobulin specific perceptions of quality of life, immunoglobulin-G

## Abstract

**Background:**

Immunoglobulin replacement therapy (IgRT) is the current standard of care for primary antibody deficiency patients (majority of all primary immunodeficiency (PID) diseases), with growing real-world evidence supporting use for secondary immunodeficiency (SID) patients. Infusion methods and practices can affect patients’ satisfaction with their treatment and perception of their health-related quality of life.

**Methods:**

An online survey of US patients with PID and SID was conducted. This research investigates primarily the impact of two IgRT infusion methods, intravenous immunoglobulin therapy (IVIG) and subcutaneous immunoglobulin (SCIG), on the patient reported outcome (PRO) Life Quality Index (LQI) tool. Patient reported infusion time efficiency, physical and mental health (PROMIS GPH-2 and PROMIS GMH-2 respectively), patient acceptability of their symptom state (PASS), upper extremity disability (Quick DASH) and general health perception (via the GHP) are also investigated.

**Results:**

Responses of 990 patients (391 IVIG and 598 SCIG) were analyzed. The median total LQI score amongst SCIG patients (84.7) was higher than IVIG patients (81.9) (*p* < 0.001), and was significantly higher on 3 out of 4 sub-domains of the LQI. SCIG patients scored higher on items that are related to convenience and reported less interference with everyday life: “Are convenient”, “Are scheduled according to my convenience”, “Do not interfere with my work/school” and “Require very little time and cost”. However, there was no significant difference between the two patient cohorts on other, non-IG specific PROs (PASS, PROMIS GPH-2 and GMH-2 and Quick DASH). Patient reported time per infusion was lower for SCIG infusions than IVIG infusions (pre-infusion time; 22 min vs. 63 min, *p* < 0.001, infusion time; 120 min vs. 240 min, *p* < 0.001, post-infusion time; 9 min vs. 31 min, *p* < 0.001). IVIG patients also reported more interference with everyday life than SCIG patients (82 vs. 86, *p* < 0.001).

**Conclusions:**

The significantly higher LQI scores for patients receiving SCIG than those receiving IVIG confirms existing evidence that substitution of SCIG for IVIG may favorably impact immunoglobulin specific perceptions of quality of life and treatment satisfaction for appropriately selected patients. Our evidence on infusion times indicates similar improvement may be possible on infusion time efficiency.

**Supplementary Information:**

The online version contains supplementary material available at 10.1186/s13223-024-00939-y.

## Background

Immunodeficiency diseases are chronic disorders that impact the functionality of the immune system and leave the individual more susceptible to infections, allergies, malignancies, and/or autoimmune diseases [1, 2]. Primary immunodeficiencies (PID) are the result of intrinsic genetic defects [1], while secondary immunodeficiencies (SID) arise due to extrinsic factors such as malnutrition, medical treatments and interventions, health conditions, and infectious diseases [2]. Antibody deficiency (hypogammaglobulinemia) can be a result of primary or secondary immunodeficiency. Despite different underlying pathologies, PID and SID are associated with similar symptoms; including recurrent, complicated, or opportunistic infections, particularly of the upper or lower respiratory tract [2, 3]. 

Treatments for PID and SID with antibody deficiency include prophylactic antibiotic therapy, immunosuppressive treatments (in those with autoimmune conditions), and immunoglobulin replacement therapy (IgRT). Ongoing IgRT with human plasma-derived immunoglobulin-G (IgG) is in fact the current standard of care for severe primary antibody deficiency, with substantial long-term evidence demonstrating reduced frequency and severity of infections [3–6]. Use of IgRT in SID patients with antibody deficiency is also growing, with increasing real-world evidence to support its use [2, 7–12]. 

Intravenous administration (IVIG) is the most widespread method of administration for IgRT. IVIG can be given in a hospital or (less frequently) in patients’ homes. IVIG infusion time ranges from 2 to 6 h depending on the total dose and individuals’ tolerance of treatment. Similarly, the frequency of IVIG administration may vary from every two, three, or four weeks depending on patient requirements and tolerance [2, 3]. 

Over the last 15 years or so, subcutaneous administration (SCIG) has been increasing in usage [13, 14]. SCIG is typically administered at home by patients [15] after learning to self-administer but can also be managed by a healthcare practitioner at home or in a healthcare setting. SCIG usually requires more frequent administration, typically weekly, of a lower dose of IgRT compared to IVIG, as subcutaneous tissue cannot accept the same volume of treatment [6]. Despite early concerns around the efficacy of SCIG, studies comparing the two administration methods found SCIG achieved acceptable IgG trough levels, had a low incidence of side effects, and similar efficacy to IVIG infusions [16, 17]. Further studies have demonstrated more stable serum IgG levels and lower incidence of systemic adverse events associated with SCIG compared to IVIG [14]. Furthermore, a meta-analysis of 946 patients with primary antibody deficiency demonstrated switching from IVIG to SCIG therapy led to higher IgG levels and fewer side effects [7, 18]. 

Despite the availability of treatments, both PID and SID significantly impact the overall health and well-being of affected individuals, leading to reduced life quality and increased morbidity. PID and SID can impact patients’ physical and emotional well-being, social interactions, family life, work productivity and cause disability which further impacts individuals’ ability to take part in normal daily living [19]. While effective treatments for PID/SID can help to relieve symptoms and reduce their associated impact, treatments themselves (including IgRT) bring their own burdens [4, 20]. In a global survey of patients with immunodeficiencies, Espanol et al. (2014) [15] found that despite receiving known effective treatments, patients’ HRQoL was below the norm for physical and mental well-being. In the same study, although most patients with immunodeficiency (76%) reported being satisfied with their treatment, more patients receiving SCIG reported being satisfied with their treatment than those receiving IVIG (83% vs. 69% respectively; *p* < 0.05). Regarding improving the treatment experience, patients receiving IgRT expressed a desire for shorter infusions, the ability to administer these at home, self-administration, and fewer needle sticks [15]. 

Since immunoglobulin infusion characteristics have been known to play an important role in lives of patients receiving IG treatments [4, 15, 21], the Life Quality Index (LQI) was designed by Daly et al. (1991) [22] to evaluate immunoglobulin specific perceptions of QOL and treatment satisfaction among patients receiving IgRT [23]. The LQI uses a Likert scale ranging from “extremely good” (= 7) to “extremely bad” (= 1) to assess 15 items related to the experience of receiving IVIG or SCIG treatment, including pain associated with treatment, side effects, convenience, and impact on health and daily living.

More generally, it is recognized that treatment administration methods impact PID/SID patients’ treatment satisfaction. A recent patient survey study that evaluated the association of IgRT administration methods on PID and SID with patient treatment satisfaction [24] (as evaluated via the TSQM-9 tool [25] concluded that patients receiving SCIG are more satisfied with their treatment relative to those receiving IVIG. Further, it was reported that the SCIG cohort was associated with a significantly higher proportion of patients in an acceptable symptom state (via PASS instrument); and with a lower proportion reporting a “very poor” or “poor” perception of their health (as measured by the General Health Perceptions tool [26]. Further, multiple longitudinal studies have reported an increase in HRQoL when switching from IVIG to SCIG [4, 20, 23]. Patient preference for SCIG can be attributed to the convenience of administering at home, reduced systemic adverse events and greater freedom and independence [13, 20]. Despite these advantages, there is also evidence that some patients may switch back to IVIG from SCIG [27, 28]. A systematic review of studies investigating the burden of IgRT on PID patients by Jones et al. (2018) [6] concluded that while patients are overall satisfied with either IVIG or SCIG, they prefer to receive treatment in their home rather than in a healthcare setting. Aside from the administration method and setting of treatment, a range of patient-specific factors could influence patients’ satisfaction with their treatment such as their job, lifestyle, and comfort level with needles [27]. 

Due to the relative equivalence in efficacy between IVIG and SCIG, it continues to be important to fully characterize and contrast specific aspects of immunoglobulin infusion-related patient experience, in the background of patient symptomatology, disability, physical and mental health, and overall general health perception of immunodeficiency patients. This research aims to understand the patient experience of receiving IgRT via different administration methods (IVIG vs. SCIG) in terms of the above aspects to guide choices that optimize immunoglobulin infusion-related quality of life and treatment satisfaction.

## Methods

### Data source

Using the Immune Deficiency Foundation’s database, patients with immunodeficiencies in the US were contacted via email regarding an incentivized online survey between April 2022 and November 2022. The survey contained 111 questions on IgRT use and respondent perceptions (Additional file 1), including demographic characteristics, reasons for choosing an IgRT infusion method, infusion characteristics, IgRT history, details of switching between IVIG and SCIG, SCIG training experiences, and structured patient-reported outcomes (PROs). PROs included (i) the Life Quality Index (LQI) [22, 23] to assess *immunoglobulin specific perceptions of quality of life and treatment satisfaction*, (ii) Patient Acceptable Symptom State (PASS) [29] to measure patient acceptability of symptom status, (iii) General Health Perception (GHP) [30] for overall health perception, (iv) the Patient-Reported Outcomes Measurement Information System (PROMIS), two-item Global Physical Health (GPH-2), two-item Global Mental Health (GMH-2) scales [31], respectively and (v) the Quick DASH [32] to assess patients’ use of the arms, shoulders, and hands and any disability of the upper extremities.

### Study exclusion criteria and study cohorts

Overall, 12,085 invites were sent out by the Immune Deficiency Foundation. 1299 respondents entered the survey, of which, 990 completed the survey and were included in the analysis (IVIG; *n* = 391, SCIG; *n* = 598). All respondents were required to have been living in the US, adults (18+), have received either SCIG or IVIG, and have either a primary or secondary immunodeficiency. Respondents were removed for incongruent responses.

### Patient reported outcomes

In this study, immunoglobulin (IgG) specific perceptions of quality of life and treatment satisfaction was the primary concept of interest, as measured by the LQI. The LQI uses a Likert scale to assess 15 items related to the experience of receiving IVIG or SCIG treatment [22, 23], including pain associated with treatment, side effects, convenience, and impact on health and daily living. The pain item was excluded from the survey since every infusion, even the subcutaneous infusions, were reported to be painful in pilot analysis and did not contribute meaningfully to differentiation of modality. In addition to evaluating the total LQI score, we also assessed the four LQI sub-domains: (i) Treatment Interference, (ii) Therapy Related Problems, (iii) Therapy Setting and (iv) Treatment Cost. LQI items were transformed to a common 0-100 scale.

Overall well-being was captured by a single-item GHP question [30]. Patient perceptions of their symptoms, current disease and health status were also of interest in this study. Patient symptom status was measured using the PASS tool [29]. 

The PROMIS GPH-2 and PROMIS GMH-2 were used to assess perceived patient physical and mental health respectively [31, 33, 34]. The Quick DASH outcome measure was implemented to assess patients’ use of the arms, shoulders, and hands and any disability of the upper extremities [32]. 

### Statistical analyses

LQI scores, GHP scores, PASS percentages, PROMIS GPH-2 and GMH-2 T-scores, and Quick DASH scores were compared overall between the IgRT infusion cohorts (IVIG vs. SCIG). Categorical variables were compared between infusion cohorts using the chi-squared test, and continuous variables were compared between groups using the Mann-Whitney test since the data was not normally distributed. All analyses were performed using the IBM SPSS version 29 software package.

In addition to testing for statistical significance, the clinical meaningfulness of each of the differences across the IVIG and SCIG groups was derived based on calculated Cohen effect sizes [33]. As the data was non-parametric, Cohen effect sizes for the LQI total score and subdomain scores were calculated as r = z/√N, where the z is the value derived by converting the Wilcoxon U statistic into a standard normal distribution using its mean and standard deviation. Effect sizes in the range 0.1–0.3 were deemed at least minimally meaningful, 0.3–0.5 moderately meaningful, and those above 0.5 highly meaningful, following standard convention [33], including in the context of the LQI in a previous study in patients with immunodeficiency [35]. 

## Results

### Respondent characteristics

In total, 990 patients with immunodeficiency receiving IgRT responded to the survey (8% of those 12,085 invited) and were included in the analysis. Of these, 391 (39%) received IVIG and 598 (61%) received SCIG.

Respondent characteristics are summarized in Table 1. The mean age of all respondents was 58.1 years with IVIG patients significantly older than SCIG patients (60.2 years and 56.8 years respectively (*p* < 0.001). There were more female patients (84%, *n* = 833) in the total cohort than male (15%, *n* = 152, *p* < 0.001). There were significantly more female respondents in the SCIG cohort compared to the IVIG cohort (87% vs. 79%, *p* < 0.001). Common variable immunodeficiency was the predominant immunodeficiency diagnosis, reported by about 3/4th of patients. Over half of patients (55%) reported some kind of permanent impairment or health-related loss, while a significantly lower proportion of SCIG users reported mobility issues than IVIG users (15% vs. 20% respectively, *p* = 0.04).

**Table 1:** Patient characteristics by IgRT administration method (total *n* = 990, IVIG *n* = 392. SCIG *n* = 598)

Respondent characteristics	Total (*n* = 990)		IVIG cohort (*n* = 392)		SCIG cohort (*n* = 598)		*P* Value
	Summary	*n*	Summary	*n*	Summary	*n*	
**Age**,** years**,** (median)**	58.1 (61)	990	60.2 (63)	392	56.8 (59)	598	< 0.001
**Age at diagnosis years**,** (median)**	46 (49)	988	47 (50)	391	45 (49)	597	NS
**Gender**,** % (n)**		990		392		598	
Female,	84% (833)		79% (310)		87% (532)		< 0.001
Male	15% (152)		21% (81)		12% (71)		< 0.001
**Specific diagnosis**,** % (n)**		990		392		598	
Common variable Immunodeficiency	74% (731)		73% (286)		74% (445)		ALL NS
Hypogammaglobulinemia	11% (104)		11% (44)		10% (60)		
Specific antibody deficiency	4% (42)		2% (8)		6% (34)		
Severe combined Immunodeficiency	1% (5)		0% (1)		1% (4)		
Combined Immunodeficiency	1% (9)		1% (2)		1% (7)		
Agammaglobulinemia	2% (19)		3% (11)		1% (8)		
IgG subclass deficiency	4% (41)		5% (19)		4% (22)		
Selective IgA deficiency	1% (7)		1% (4)		1% (3)		
Secondary Immune Deficiency	3% (27)		4% (15)		2% (12)		
**Patient health impairments or loss status**,** % (n)**		970		380		590	ALL NS EXCEPT mobility
No permanent losses	45% (438)		41% (157)		48% (281)		
Lung function	22% (217)		22% (83)		23% (134)		
Digestion	21% (201)		23% (87)		19% (114)		
Hearing	18% (175)		20% (77)		17% (98)		
Mobility	17% (165)		20% (77)		15% (88)		= 0.04
Vision	15% (142)		16% (59)		14% (83)		
Neurological function	12% (116)		14% (54)		11% (62)		
Kidney function	5% (52)		6% (24)		5% (28)		
Liver function	4% (43)		6% (21)		4% (22)		
Hand/eye coordination	4% (39)		5% (20)		3% (19)		
Other	9% (91)		12% (46)		8% (45)		

### IgRT infusion characteristics

The most common IVIG infusion interval was every 4 weeks (62%), ranging from weekly (or more frequently) to every 5 weeks or more. 46% of patients had received an infusion within the last 2 weeks.

Over half of IVIG patients indicated that their clinician had had difficulty in finding a vein while administering IVIG (52%). Of these patients, 25% experienced an interruption to treatment and had to reschedule their appointment. Further, 25% of the IVIG patients who faced an interruption to treatment required subsequent use of a central venous access device (CVAD).

The home setting was the most common for IVIG infusions, with 47% of IVIG patients receiving infusions in this setting, followed by 37% who reported attending an infusion center to receive IVIG, and 11% who received it in a hospital. The mean travel time to the IVIG infusion setting was 70 min, ranging from less than 15 min (7% of IVIG patients who travel for infusions) to more than 1.5 h (20% of IVIG patients who travel for infusions).

Over half (53%) of SCIG users had received IVIG prior to SCIG. Of these, 89% switched to their current SCIG method directly from IVIG and 11% switched from another SCIG method. All SCIG patients self-administered their doses. For the initial training, 80% were trained at home, and 7% at an infusion center; and 85% had between one and three total training sessions. 64% of SCIG patients reported they receive their infusions weekly, while only 8% reported more frequent infusions and 28% reported receiving less frequent infusions every 2–4 weeks.

For IVIG patients, the mean time spent preparing for an infusion (pre-infusion time, including check-in, waiting and preparation) was 63 min (median = 30 min) (see Table 2); 64% of IVIG patients reported a pre-infusion time of 30 min or less. The mean pre-infusion time per infusion for SCIG users was significantly shorter at 22 min per typical weekly infusion (*p* < 0.001) (median = 18 min) (see Table 2), and 50% of SCIG patients reported a pre-infusion time of 15 min or less. The majority of SCIG patients (66%) received an infusion every week, while most IVIG patients (62%) received an infusion every 4 weeks. For IVIG patients, the mean infusion time was 4 h (median = 3 h 38 min) (see Table 2); nearly a quarter (24%) reported infusion times from 4 h to 4 h 59 min and a further 24% reported infusion times of 5 h or more. For SCIG patients, the infusion time was significantly shorter with a mean infusion time of 2 h (median = 1 h 45 min) (*p* < 0.001) (see Table 2), and 57% of SCIG patients reported an infusion time of 1 h 59 min or less.

For IVIG patients, the mean post-infusion time (including clean-up and waiting to check out) was 31 min (median = 10 min) (see Table 2), and 94% of patients reported a post-infusion time of 30 min or less. The mean post-infusion time for SCIG patients was significantly shorter than IVIG patients at 9 min (median = 8 min) (*p* < 0.001) (see Table 2), and 92% of patients reported a post-infusion time of 15 min or less.

**Table 2:** Average time (mean) for preparation before each infusion, infusion time, and post-infusion time for IVIG and SCIG. (IVIG *n* = 376, SCIG *n* = 598)

	IVIG (*n* = 376)	SCIG (*n* = 598)	*P* value
**Preparation time before each infusion (minutes**			
Mean score	63.40	22.32	*p* < 0.001
Standard deviation	100.83	15.85	
**Infusion time (minutes)**			
Mean score	237.77	119.07	*p* < 0.001
Standard deviation	102.63	105.70	
**Post-infusion time (minutes)**			
Mean score	31.27	9.04	*p* < 0.001
Standard deviation	182.78	6.08	

Note: table based on available data for *n* = 376 out of a total IVIG *n* = 392 patients

### Patient-reported outcomes

#### Life quality index

**Fig. 1:**
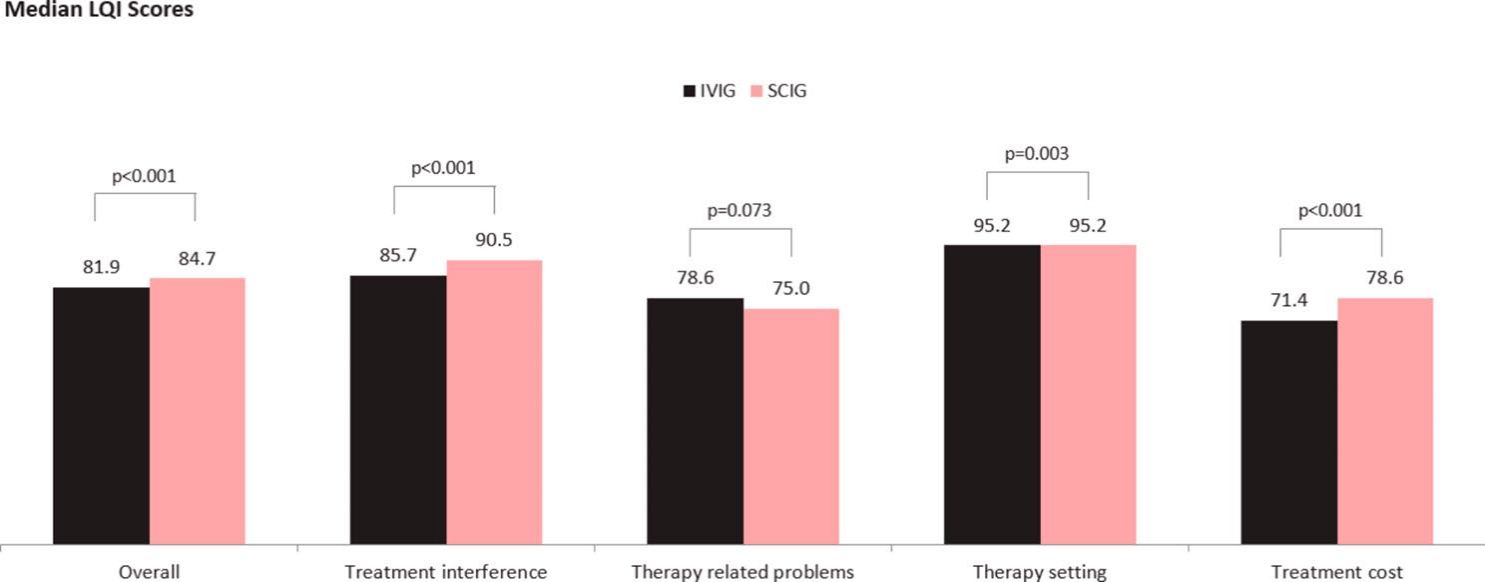
Median LQI Scores – Total and by LQI sub-domain: IVIG vs. SCIG. Note: figure based on available data for *n* = 376 out of a total IVIG *n* = 392 patients and *n* = 598 SCIG patients. The inferential p-values are based on the full distribution, and not on medians alone

The median LQI score amongst SCIG users was statistically significantly higher than for IVIG users (84.7 vs. 81.9) (mean 82.2 (SD = 11.1) vs. 79.5 (SD = 12.2), respectively) (Fig. 1), (*p* < 0.001), with an effect size of 0.11 (minimally meaningful). Further the median LQI score amongst SCIG users was also significantly higher on 3 out of 4 sub-domains of the LQI – Treatment Interference (90.5 vs. 85.7) (mean 86.1 (SD = 13.3) vs. 82.1 (SD = 14.7), respectively) (effect size 0.14, minimally meaningful), Therapy Setting (95.2 vs. 95.2) (mean 91 (SD = 12.3) vs. 87.6 (SD = 15.4), respectively) (effect size 0.10, minimally meaningful), and Treatment Cost (78.6 vs. 71.4) (mean 74.3 (SD = 18.9) vs. 68.1 (SD = 22.5), respectively) (effect size 0.13, minimally meaningful) (Fig. 1**).** There were no statistically significant nor clinically meaningful (effect size 0.06) difference between the two groups on the sub-domain of Therapy-related Problems (median 75 SCIG vs. 78.6 IVIG) (mean 73.9 (SD = 12.6) SCIG vs. 75.2 (SD = 12.6) IVIG).

**Fig. 2:**
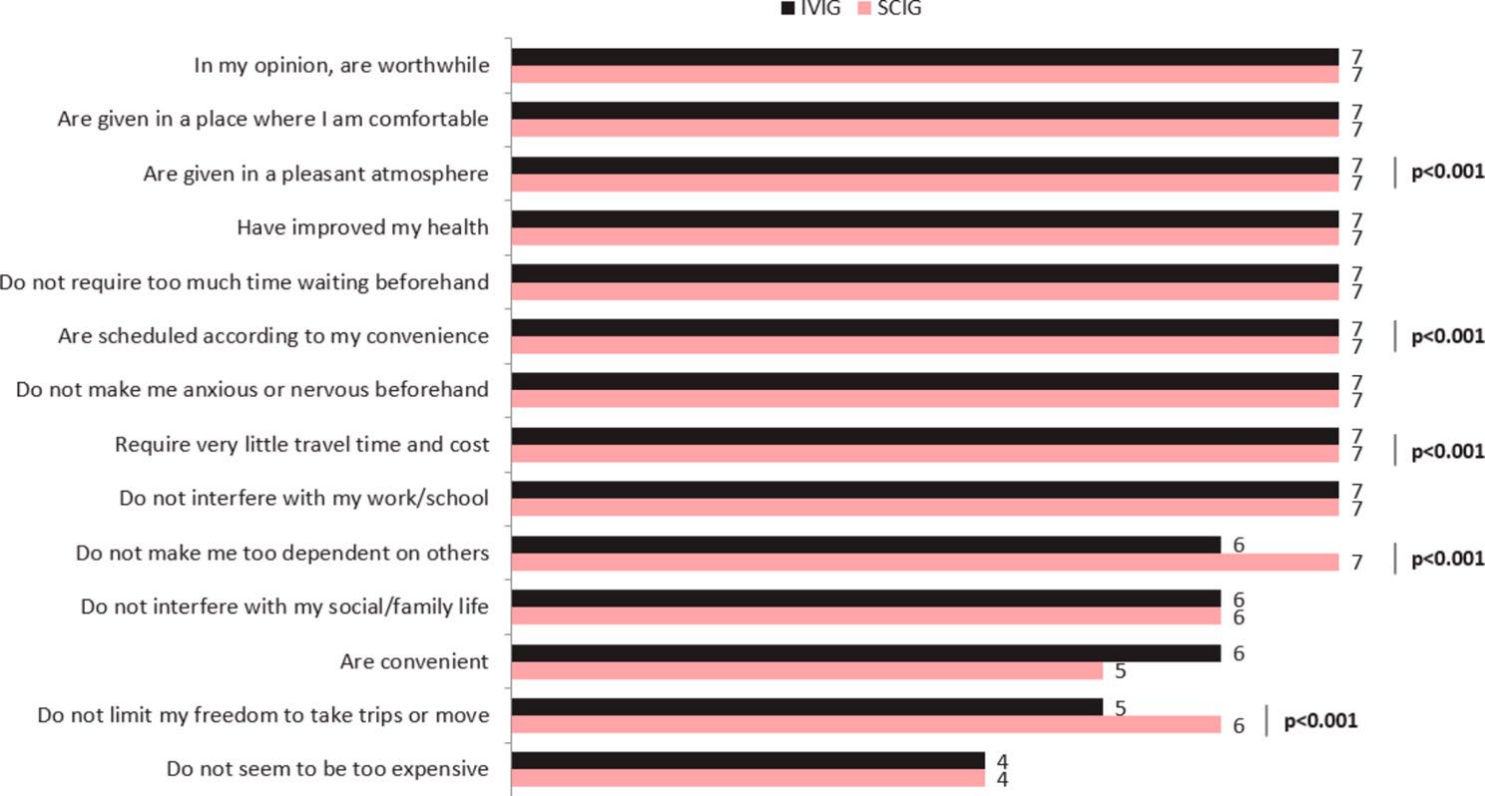
Item/ attribute specific LQI median scores: IVIG vs. SCIG. Note: IVIG *n* = 392, SCIG *n* = 598. The inferential p-values are based on the full distribution, and not on medians alone

In addition to summary total LQI and LQI sub-domain scores, we also evaluated item specific outcomes. This was assessed in two ways – in terms of (a) item specific scores and (b) proportion of patients achieving the top two score levels on each item. Figure 2 presents item specific scores, comparing the IVIG and SCIG groups. These reveal that the SCIG group had numerically better or similar scores to the IVIG group on all items but significantly better scores on the items “Are given in a pleasant atmosphere” (mean 6.83 median 7, vs. mean 6.54 median 7, respectively) (*p* < 0.001), “Are scheduled according to my convenience” (mean 6.65 median 7, vs. mean 6.13 median 7, respectively) (*p* < 0.001), “Require very little travel time and cost” (mean 6.6 median 7, vs mean 5.83 median 7, respectively) (*p* < 0.001), “Do not make me too dependent on others” (mean 5.81 median 7, vs mean 5.2 median 6, respectively) (*p* < 0.001), and “Do not limit my freedom to take trips or move” (mean 5.21 median 6, vs mean 4.55 median 5, respectively) (*p* < 0.001).

**Fig. 3:**
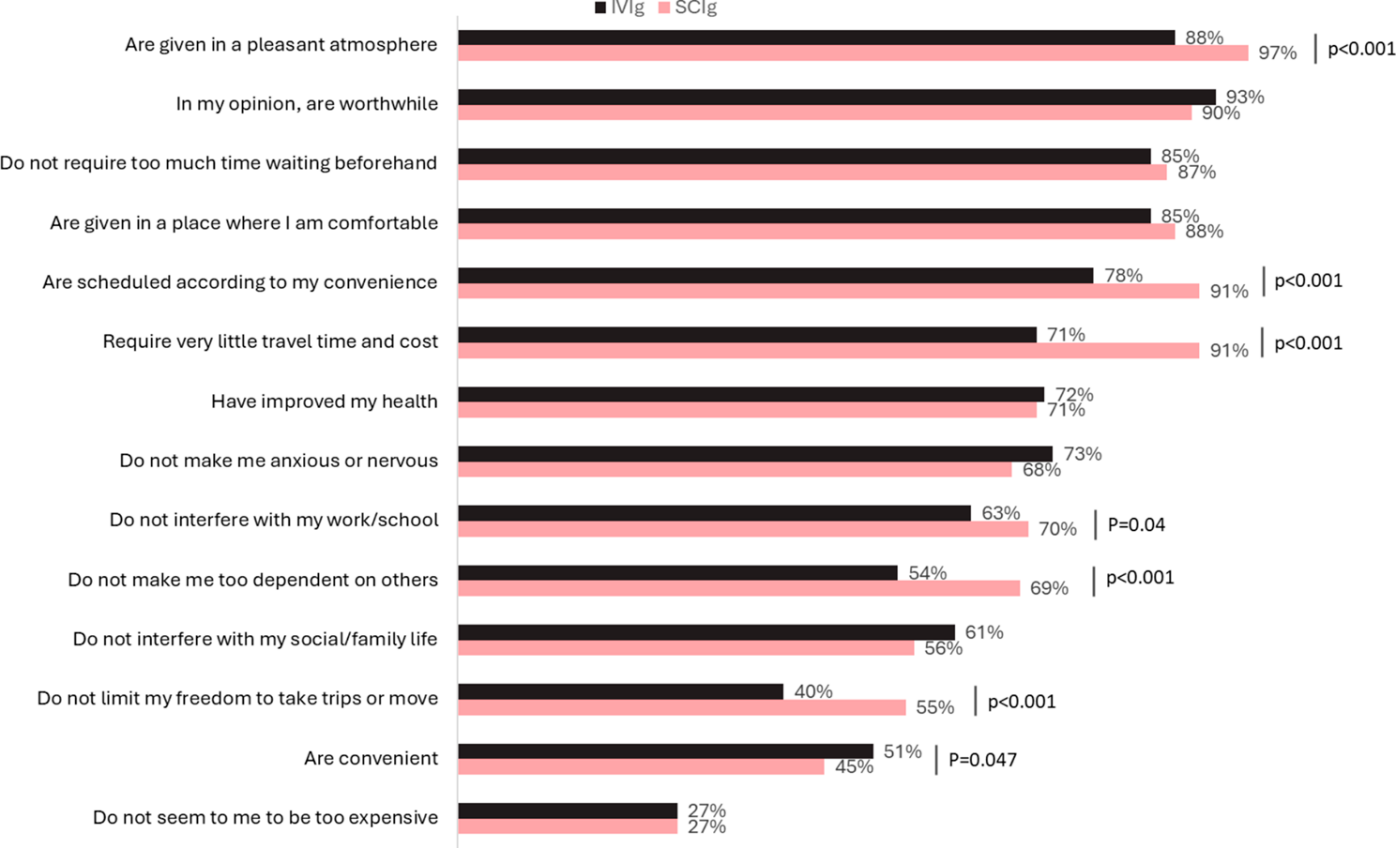
Percentage of respondents scoring their IgRT in the top two scores (6 or 7) for each LQI item (attribute)

When comparing item-specific scores in terms of the proportion of patients who achieved the top two scores (6 or 7), the SCIG group had significantly higher proportions than IVIG patients achieving top two score levels for the attributes: “Are given in a pleasant atmosphere” (97% vs. 88%, *p* < 0.001), “Are scheduled according to my convenience” (91% vs. 78%, *p* < 0.001), “Require very little time and cost” (91% vs. 71%, *p* < 0.001), “Do not interfere with my work/school” (70% vs. 63%, *p* = 0.04), “Do not make me too dependent on others” (69% vs. 54%, *p* < 0.001) and “Do not limit my freedom to take trips or move” (55% vs. 40%, *p* < 0.001) (see Fig. 3).

Finally, on the LQI, we evaluated scores by stratifying IVIG patients in terms of whether they received their IVIG infusions (a) at home or (b) non-home settings (IVIG infusion centers or hospital outpatient center). Mirroring results for the overall evaluation, the SCIG group, when compared to the IVIG group that received non-home based infusions, had significantly better total LQI scores and on three of four LQI sub-domains, except the Therapy-related Problems sub-domain. When compared to the IVIG home infusion group, the SCIG group had significantly better scores on the Treatment Interference sub-domain alone, while LQI scores were not significantly different in total or the other three sub-domains.

### Current health and disease status

The mean PROMIS PH2A T-score (assessing patient reported physical health) was 42.8 with a range of 23.4 to 63.3. There was no significant difference between the scores of IVIG and SCIG patients (42.8 and 42.9 respectively). Overall, 71% of patients had PROMIS GPH-2 T-score of less or equal to 45 (1/2 SD or more below population norm of 50), thus scored meaningfully (1/2 SD or more) below the population norm. This proportion was 73% for IVIG and 70% for SCIG patients. The mean PROMIS GMH-2 T-score (assessing patient reported mental health) for all patients was 45.8 with a range of 25.8 to 64.6 with no significant difference between the scores of IVIG and SCIG patients (46.1 and 45.6 respectively). Overall, 59% of patients on PROMIS PH were at scores meaningfully (1/2 SD or more) below the population norm (score of 45). This was 57% for IVIG and 60% for SCIG patients.

### Impact of symptoms on daily activities

Overall, 18% of patients reported that work or school was affected by their IgRT (Table 3). IVIG patients were significantly more likely to miss school or work than SCIG patients (28% vs. 11% respectively, *p* < 0.001). 34% of patients reported losing half to a full day of work or school per month, a further 18% reported missing 1 to 2 days per month, and 24% reported missing more than two days per month. IVIG patients were significantly more likely to report that they typically lose half or full day of work/school than SCIG patients (44% vs. 20%, *p* < 0.001).

**Table 3:** Impact of immunoglobulin infusions on work or school^1^

“Does any part of the treatment regimen cause you to miss work/school?”	Total	IVIG	SCIG	*P* value
Base: All respondents	(*n* = 990)	(*n* = 392)	(*n* = 598)	
Yes	18%	28%	11%	< 0.001
No	82%	72%	89%	
***“How much time of work/school per month do you typically miss?”***				
Base: Those who miss work/school	(*n* = 174)	(*n* = 108)	(*n* = 66)	
Less than 1 h	2%	0%	6%	NS
Between 1–3 h	11%	10%	14%	NS
Half a day to one full day	34%	44%	20%	< 0.001
1–2 days	18%	20%	15%	NS
More than 2 days	24%	20%	30%	NS
Not sure	9%	6%	15%	NS

^1^Responses to the question “Does any part of the treatment regimen cause you to miss work/school?“. Those who responded “yes” were asked the follow-up question “How much time of work/school per month do you typically miss?”

Note Total *N* = 990, IVIG *n* = 392, SCIG *n* = 598)

The average Quick DASH (measure assessing patients’ use of the arms, shoulders, and hands and disability of the upper extremities) score was 23.5 with no significant difference between IVIG and SCIG patients.

Quick DASH scores were low across all attributes (see Table 4). The highest scoring attribute was “Interference with normal social activities”, where 10% of respondents indicated that their arm, shoulder, or hand pain interfered with normal social activities “quite a bit” or “extremely”. Significantly more IVIG users than SCIG users indicated that their arm, shoulder, or hand pain interfered “quite a bit” or “extremely” in normal social activities (14% vs 8%, *p* = 0.009).

**Table 4:** Percentage of respondents scoring their IgRT in the top two scores (the lowest level of limitation or impediment on ability in terms of arm, shoulder, or hand problems/pain) for each quick DASH item

Quick DASH survey instrument items	Total (*n* = 990)	IVIG patients (*n* = 392)	SCIG patients (*n* = 598)	*P* value
Interference with normal social activities	10%	14%	8%	= 0.009
Limited work or other regular daily activities	8%	10%	7%	NS
Severity of pain	6%	7%	6%	NS
Severity of tingling	4%	5%	4%	NS
Difficulty sleeping	3%	3%	4%	NS

Note Total *N* = 990, IVIG *n* = 392, SCIG *n* = 598

## Discussion

In this analysis of a survey among patients with various immunodeficiencies, the association of IgRT administration method with patient-reported outcomes was evaluated by comparing the responses of the cohort receiving IVIG and the cohort receiving SCIG.

Overall, common variable immunodeficiency was the predominant type of immunodeficiency diagnosis, reported by about 3/4th of patients followed by general hypogammaglobulinemia, reported by an additional 11%. Other immunodeficiencies were reported much less frequently, including secondary immunodeficiency (SID) reported by 3%. SCIG was the more common of the 2 modes of IG administration, reported by 60% of patients, reflective of its growing role in treatment [24], including as high as 40% in patients with SID, consistent with another recent survey [24]. Patients receiving SCIG, compared to those on IVIG, reported a significantly greater score on the LQI tool. This finding is consistent with previous studies that have evaluated the relationship between IG administration methods and patient reported outcomes including quality of life or treatment burden. Gardulf et al. (2008) [4] found that a weekly SCIG infusion regimen was associated with significant improvements in HRQoL and treatment satisfaction, and as noted earlier, particularly in patients who had previously received IVIG therapy in hospital settings. A report on 6 clinical studies conducted in the US, Japan and Europe revealed significant improvements in IgG specific perceptions of quality of life and treatment satisfaction, as measured by the LQI, and in physical function and general health based on corresponding domains of the SF-36v2 Health Survey [35]. A recent study by Kan et al. (2022) [36] also reported higher health status scores (measured by SF-36v2 Health Survey) and IgG specific perceptions of quality of life and treatment satisfaction (LQI) among SCIG users compared to IVIG users. Finally, a recent survey of immunodeficiency patients in Quebec, Canada [24] found that patients on SCIG were associated with higher scores on the effectiveness domain of the Treatment Satisfaction and Quality Medication PRO tool [25], as also with greater acceptability of their symptom state, measured by the Patient Acceptability of Symptom State (PASS) tool [29]. 

Reported results on the LQI sub-domains and items shed additional light on specific perceived differences between the IVIG and SCIG groups. Thus, while the SCIG group was associated with better total and better 3 out of 4 sub-domain scores, the only exception was the Therapy-Related Problems sub-domain which contains three items, one of which refers to IG treatments in terms of whether they “Have improved my health”, arguably not surprising since IG modes of administration are not expected to differ in terms of their therapeutic effectiveness. On the other hand, SCIG users were significantly more likely to give one of the top two positive scores (6 or 7) on LQI items related to convenience (part of the LQI Treatment Interference sub-domain) including “Are scheduled according to my convenience”, “Do not make me too dependent on others” and “Do not limit my freedom to take trips or move”, and accordingly suggest that SCIG users perceived their treatment to be less lifestyle-limiting than IVIG users. Additionally the finding that SCIG users reported significantly higher scores as well as a higher percentage reporting top two score levels on an item/attribute such as (my IG treatments) “Are given in pleasant atmosphere” (part of the LQI Therapy Setting sub-domain) suggests that SCIG patients find their treatment setting, always their home, to be more pleasant, than IVIG patients find their treatment setting, typically although not exclusively, an infusion center/hospital, to be. Finally, the item (My IG treatments) “Require very little time and cost” was scored significantly higher (better) for SCIG users (as was the Cost sub-domain to which the item belongs) which also suggests that IVIG administration related (unreimbursed) travel costs and time burden does seem to have an unfavorable impact on patients receiving IVIG, compared to SCIG, consistent with evidence on the burden of IV infusions in other conditions as well [37–39]. Together, these factors contribute most to differences in IgG specific perceptions of quality of life and treatment satisfaction, as measured by the LQI.

In our evaluation of the LQI stratified by whether or not IVIG patients received their infusions at home or in the infusion center/hospital outpatient setting, we found that the latter sub-group of IVIG patients reported significantly more unfavorable LQI outcomes than IVIG patients receiving home infusions when each was compared to the SCIG group. Yet, even IVIG patients receiving home infusions had significantly poorer scores, compared to SCIG patients, on the Treatment Interference sub-domain which includes items such as (my IG treatments) “limiting my freedom to take trips or move” and “interfere with my social/family life” suggesting that health-care professional administered home IVIG infusions still limit patients’ freedom and sense of privacy and self-control. Overall however, these differences in LQI scores, by setting of IVIG administration, are consistent with a study by Kearns et al. (2017) [5] that did not find a significant difference between IVIG and SCIG users in the individual attribute “are convenient”, as all IVIG patients received their IgRT in the home setting, which is not typical [21, 24]. Consistent with Gardulf et al. (2008) [4], these findings seem to suggest that non-home setting of IVIG infusions certainly exacerbates the greater inconvenience of IVIG administration relative to SCIG administration.

SCIG users also scored other items on the LQI Treatment Interference sub-domain higher than IVIG users. Ratings were higher for SCIG users on the following LQI items: “Do not interfere with my work/school”, “Do not make me too dependent on others”, and “Do not limit my freedom to take trips or move”. Similarly, Lechanska-Helman et al. (2020) [40] found that where parents preferred SCIG over IVIG for children with antibody deficiencies, this choice was driven by the comparatively lower interference with their work or children’s schooling.

Finally, the two LQI items that assess treatment ‘effectiveness’, “My IG treatments, in my opinion are worthwhile” and “Have improved my health” were neither numerically nor statistically different between the IVIG and SCIG cohorts, reinforcing what would be expected in terms of similar treatment effectiveness of the two modes of administration. Taken together, these findings suggest it is convenience and the lack of interference with everyday life) that contribute to the higher overall IgG specific perceptions of quality of life and treatment satisfaction of SCIG patients with immunodeficiency.

Separately from LQI responses, IVIG users were significantly more likely to report missing work or school than SCIG users, indicating a potentially greater interruption to daily life due to treatment. This finding is supported by a previous systematic review and meta-analysis of home-based SCIG versus hospital based IVIG in treatment of primary antibody deficiencies by Abolhassani et al. (2012) [17], which found that home-based SCIG treatment was associated with fewer missed days of work. Lechanska-Helman et al. (2020) [40] similarly observed greater absence from school or work associated with IVIG use than SCIG use.

Overall, the Quick DASH score was low for both IVIG and SCIG users, indicating a low rate of disability/difficulty in the use of the shoulders and upper extremities. However, analysis of the individual scores indicated that interference with normal social activities was the highest scoring element (10% said that their arm, shoulder, or hand pain interfered “quite a bit” or “extremely” in normal social activities). IVIG patients were significantly more likely to report this interference and may have been selected for IVIG infusions on account of this greater upper-extremity disability that may be problematic with SCIG self-infusions. The IVIG cohort was also older than the SCIG cohort (60.2 years (63) vs. 56.8 years (59), (*p* < 0.001) respectively), and greater disability/difficulty in the use of the shoulders and upper extremities is expected in an older population.

Similarly, PROMIS PH-2 and MH-2 scores, measuring “physical health-” and “mental health-related function”, respectively, were not significantly different across the IVIG and SCIG users, which is not surprising, as in a cross-sectional survey comparing two cohorts, several external unmeasured factors can influence such general health outcomes [40]. This was also evident in the similarity on patient acceptability of current symptoms as measured by PASS and overall health perceptions (GHP). As previously noted, by contrast, longitudinal studies of switch from IVIG to SCIG have demonstrated improvements with change even in terms of physical function (as measured by SF-36 v2) [35]. 

The likelihood of missing school or work was found to be higher for patients on IVIG infusions. This is not surprising since all stages of IgRT took longer; the mean total time investment per infusion for IVIG was more than twice that of each SCIG infusion exclusive of travel time. While the majority of SCIG patients must infuse more regularly than IVIG users (weekly vs. every 4 weeks), it is shorter durations per infusion that have previously been demonstrated to improve patient satisfaction with treatment [15, 21]. Ultimately, at a patient level, choices between the two modalities could allow treatment regimens to be tailored to patient lifestyle and preference for either the more frequent but shorter infusions or longer but less frequent infusions. In summary, our results indicate that ideally switching from IVIG to SCIG would be expected to improve treatment satisfaction on a number of dimensions, but if for patient specific reasons such as lack of comfort with self-infusions, patients are unable to switch to SCIG, then at minimum switching IVIG patients to home infusion and/or more patient-sensitive scheduling of infusions would certainly be of potential patient benefit.

It is important to interpret PRO findings not only in terms of statistical significance but also clinical meaningfulness, in terms of anchor-based and/or empirical methods [41–43]. No published studies have evaluated clinically meaningful differences on the LQI in terms of a clinical anchor. Yet, clinical meaningfulness of observed change in the LQI has been determined empirically in terms of the Cohen effect size in a pooled analysis of phase 3 clinical studies evaluating switch from IVIG to SCIG in PID (Mallick et al. 2018). In our cross-sectional study, we similarly deduced clinical meaningfulness based on evaluated effect sizes and determined that differences between the IVIG and SCIG groups on the overall LQI, the Treatment Interference subdomain, the Therapy Setting subdomain, and the Treatment Cost subdomain were all minimally meaningful; however, the difference on the Therapy-related Problems subdomain was determined to be not meaningful. The effect sizes in our study were somewhat lower than those in Mallick et al. 2018, which also found minimal meaningfulness on the overall LQI but also all subdomain scores including the Therapy-related Problems subdomain, that was determined to not be meaningful in our study. The larger LQI improvements with patients switching from IVIG to SCIG, as in Mallick et al. 2018 compared to IVIG-SCIG differences across patient groups, identified in this study may be attributable to different study designs with respect to patient selection. The clinical studies presumably permitted an opportunity for patients switching from IVIG to benefit from previously unavailable SCIG, while in our observational survey, across-group IVIG vs. SCIG differences are likely to reflect in part that patients may already be optimized to their mode of administration based on real world preference or physician selection [44, 45]. Certainly the greater reported upper extremity disability in the IVIG group reflecting in our study would seem to suggest these patients may have been found unsuitable for self-infused SCIGs [44, 45]. 

### Study limitations

There are inherent limitations with patient-reported surveys, as with any real-world data analysis, which should be borne in mind in interpretation of the evidence. Survey responses inherently rely on patient understanding of the survey questions as unlike in-patient interviews, there is not an opportunity to clarify their meaning. Further, responses to scales assessing various concepts can be contextualized by patients in different ways depending on individual personality and disposition (optimism versus pessimism, happiness versus unhappiness) [46]. Such influences are however more likely to affect the general rather than specific concepts and measurements, as for example suggested by the Wilson-Cleary framework of health outcomes; [47] thus in our study more likely to be reflected in assessment of general health perceptions than immunoglobulin specific perceptions of quality of life and treatment satisfaction [21]. Additionally, as survey responses were not independently verified with patients’ physicians, some of the findings especially as relates to specific immunodeficiency subtype and/or other related conditions may be impacted by absence of fully accurate patient recall on their medical history [48]. Due to the nature of rare disease research, the survey had a relatively low response rate and may have been skewed in terms of certain respondent groups, for example immunodeficiency sub-type. Thus, although IG is known to be underutilized among patients with SID [9, 10], one might still have expected a somewhat higher share of SID among the immunodeficiency population receiving IG therapies in this survey, as another recent survey revealed an overall SID proportion of 10% among IG patients with immunodeficiencies [24], and to that degree results in this survey may not be generalizable to SID. Finally, patient perceptions and preferences for modes of administration can change over time, which was not able to be captured in this cross-sectional survey.

Only US patients were included in this study, and therefore findings may not generalize to outside the US due to practical differences in how patients can access Ig. For example, in the US the availability of home healthcare services permits the opportunity to administer IVIG at home, which is not an option in all countries. Similarly, SCIG can also be collected in the US from local specialty pharmacies or delivered at home, adding to SCIG convenience that may not be possible in other countries where SCIG may have to be collected, for example from specialized blood banks or hospital pharmacies, and may take an average of an hour or more each time [24]. 

## Conclusion

Our study found that patients with PID or SID receiving SCIG treatment had significantly higher LQI scores than those receiving IVIG, indicating infusion method can favorably impact immunoglobulin specific perceptions of quality of life and treatment satisfaction for these patients. This is not surprising given our additional finding that SCIG requires a lesser time investment per infusion, and which has been previously shown to improve patient satisfaction with treatments notwithstanding an increase in administration frequency [15, 21]. The variation in infusion characteristics means that choice between the two modalities could allow treatment regimens to be tailored to patient lifestyle and improve treatment satisfaction.

## Electronic supplementary material

Below is the link to the electronic supplementary material.

Supplementary Material 1

## Data Availability

The datasets analysed during the current study are available from the corresponding author on reasonable request.

## Abbreviations

CVAD: Central venous access device
GHP: General health perception
HRQoL: Health-related quality of life
IgG: Immunoglobulin G
IgRT: Immunoglobulin replacement therapy
IVIG: Intravenous immunoglobulin
LQI: Life Quality Index
PASS: Patient acceptable symptom score
PROs: Patient reported outcomes (PROs)
PROMIS: Patient-reported outcomes measurement information system
PID: Primary immunodeficiency
PROMIS GMH-2: Patient-Reported Outcomes Measurement Information System two-item Global Mental Health
PROMIS GPH-2: Patient-Reported Outcomes Measurement Information System two-item Global Physical Health
QoL: Quality of life
Quick DASH: Quick disabilities of arm, shoulder or hand
SID: Secondary immunodeficiency
SCIG: Subcutaneous immunoglobulin
TSQM-9: Treatment satisfaction questionnaire for medication (9 item version)
