# Supplementary material for: Immunoglobulin replacement therapy in patients with primary and secondary immunodeficiencies: impact of infusion method on immunoglobulin-specific perceptions of quality of life and treatment satisfaction
Source: Allergy Asthma Clin Immunol. 2025 Jan 7;21:2. doi: 10.1186/s13223-024-00939-y (PMC11706029; doi:10.1186/s13223-024-00939-y)
Supplement: Supplementary file 1 — Supplementary Material 1 [file 13223_2024_939_MOESM1_ESM.pdf]

Immune Deficiency Foundation

The Immune Deficiency Foundation (IDF) is asking members from its database if they would be willing to participate in a survey about their experiences with immunoglobulin (Ig) replacement therapy.

We specifically need the participation of adults diagnosed with either a primary immunodeficiency (PI) or secondary immunodeficiency (SID) who currently reside in the United States.

The survey is being conducted as an online survey for one of the manufacturers of immunoglobulin and this manufacturer has asked IDF to help recruit survey participants for this survey. IDF is responsible for the programming of the survey. IDF does not and will not give or sell patient contact information to any third party, which is why you are receiving this e-mail directly from IDF and not from the manufacturer.

The survey has two goals:

- Help the Ig therapy manufacturer better understand our community's experiences with Ig replacement therapy.

- Help the manufacturer better understand Ig replacement therapy users' experiences to provide patients with better offerings in terms of modes of administration and IG drug packaging options and improve patient experiences.

The entire survey should, depending on your answers, take approximately 30-35 minutes to complete.

We do our best to keep your information confidential. Survey data is obtained and transmitted through a secure and encrypted process. All your answers are grouped with all the other answers, with none of your personal identifying information reported or used in the reporting.

It is possible that de-identified data obtained from this survey might be shared with other researchers or organizations conducting primary immunodeficiency research. If this occurs, all personally identifying information will be removed in accordance with HIPPA regulations. The purpose of such data sharing is to aid future research on patients' experiences.

We and our pharmaceutical manufacturer sponsor are committed to ensuring the safety of patients receiving pharmaceutical treatments. As you may know, the law also requires reporting of adverse events associated with the use of treatments to appropriate health authorities. Accordingly, we are required by these clients to pass on to them information on adverse events potentially associated with treatments that are shown during market research surveys. As a general matter, we treat your answers as confidential to interviews, as already noted above. However, if you provide information regarding an adverse event, in that case alone, in the interest of patient safety, we will report this to our client's Product Surveillance group.

Your participation in the survey is completely voluntary. Refusal to participate will not involve a penalty or loss of benefits to which you are entitled from IDF, and you may refuse to answer questions or discontinue your participation at any time.

Incentives

If you complete the survey, you will have the opportunity to enter a drawing for the chance to win an Amazon Coupon Code. Three individuals who complete the survey will be randomly selected for a chance to win one of three coupon codes in the amounts of $300, $200, and $100.

If you have any questions or concerns about this research, or your rights as a survey participant, please contact Christopher Scalchunes, Vice President of Research at IDF. He can be reached at: 1.800.296.4433.

* 1. ELECTRONIC CONSENT: Please select your choice below. Clicking on the "agree" button below indicates that:

-you have read the above information
-you voluntarily agree to participate
-You currently reside in the United States
-you are at least 18 years of age (you must be at least 19 years of age if you live in Alabama or Nebraska).

If you do not wish to participate in this survey, please decline participation by clicking on the "Disagree” button.

- ◯ Agree
- ◯ Disagree

* 2. Please select the answer that most closely describes you.

- ◯ Adult with PI
- ◯ Parent/Caregiver of a child with PI
- ◯ Both an adult with PI and a parent/caregiver of a child with PI
- ◯ Adult with SID
- ◯ Parent/Caregiver of a child with SID
- ◯ Both an adult with SID and a parent/caregiver of a child with SID
- ◯ Neither

If you are both a person with PI/SID and the parent of a child with PI or SID, **please only answer the questions as they relate to you**.

3. Are you registered with the Immune Deficiency Foundation?

- Yes
- No
- I Don't know

* 4. What is your current age?

Immune Deficiency Foundation

5. How old were you when you were diagnosed with a PI or SID? (Please specify in years)

6. What is your gender

- Male
- Female
- Other
- Refused

7. What is your current weight in pounds?
(we need this to calculate your Ig therapy dosing)

8. What is your specific PI Diagnosis?
PLEASE SELECT ONLY ONE

- Agammaglobulinemia (XLA)
- Chronic Granulomatous Disease
- Common Variable Immunodeficiency
- Combined Immunodeficiency
- Complement Deficiency
- DiGeorge Syndrome
- Hereditary Angioedema
- Hyper IgE Syndrome
- Hyper IgM Syndrome
- Other (please specify)
- Hypogammaglobulinemia
- IgG Subclass Deficiency
- Secondary Immunodeficiency (SID)
- Selective IgA Deficiency
- Severe Combined Immunodeficiency
- Specific Antibody Deficiency
- Wiskott-Aldrich Syndrome
- Not sure

9. In general, would you say your health is:

- Very Poor
- Poor
- Fair
- Good
- Very Good
- Excellent

* 10. Have you ever been treated with IgG treatment (intravenous immunoglobulin (IVIg) or subcutaneous immunoglobulin (SCIg) or other IgG treatment)?

- Yes
- No

Immune Deficiency Foundation

* 11. Are you currently being treated with IgG treatment (IVIg, SCIg or other IgG treatment)?

- ◯ Yes
- ◯ No

12. Why are you no longer being treated with IgG treatment (IVIg, SCIg or other IgG treatment)?

- ◯ Treatment discontinued by physician due to disease improvement
- ◯ Side effects
- ◯ Lack of insurance coverage
- ◯ No longer needed
- ◯ I don't know/Not sure
- ◯ Other (please specify)

The following questions will ask about your symptoms, as well as your ability to perform certain activities.

Please answer every question, based on your condition over in the last week, by selecting the appropriate number.

If you did not have the opportunity to perform an activity in the past week, please make your best estimate of which response would be the most accurate.

It doesn't matter which hand or arm you use to perform an activity; please answer based on your ability regardless of how you perform the task.

13. Open a tight or new jar.

- NO DIFFICULTY
- MILD DIFFICULTY
- MODERATE DIFFICULTY
- SEVERE DIFFICULTY
- UNABLE

14. Do heavy household chores (e.g., wash walls, floors).

- NO DIFFICULTY
- MILD DIFFICULTY
- MODERATE DIFFICULTY
- SEVERE DIFFICULTY
- UNABLE

15. Carry a shopping bag or briefcase.

- NO DIFFICULTY
- MILD DIFFICULTY
- MODERATE DIFFICULTY
- SEVERE DIFFICULTY
- UNABLE

16. Wash your back.

- NO DIFFICULTY
- MILD DIFFICULTY
- MODERATE DIFFICULTY
- SEVERE DIFFICULTY
- UNABLE

17. Use a knife to cut food.

- NO DIFFICULTY
- MILD DIFFICULTY
- MODERATE DIFFICULTY
- SEVERE DIFFICULTY
- UNABLE

18. Recreational activities in which you take some force or impact through your arm, shoulder or hand (e.g., golf, hammering, tennis, etc.).

- NO DIFFICULTY
- MILD DIFFICULTY
- MODERATE DIFFICULTY
- SEVERE DIFFICULTY
- UNABLE

19. During the past week, *to what extent* has your arm, shoulder or hand problem interfered with your normal social activities with family, friends, neighbors or groups?

- ◯ NOT AT ALL
- ◯ SLIGHTLY
- ◯ MODERATELY
- ◯ QUITE A BIT
- ◯ EXTREMELY

20. During the past week, were you limited in your work or other regular daily activities as a result of your arm, shoulder or hand problem?

- ◯ NOT LIMITED AT ALL
- ◯ SLIGHTLY LIMITED
- ◯ MODERATELY LIMITED
- ◯ VERY LIMITED
- ◯ UNABLE

21. Please rate the severity of the following symptoms in the last week: Arm, shoulder or hand pain.

- ◯ NONE
- ◯ MILD
- ◯ MODERATE
- ◯ SEVERE
- ◯ EXTREME

22. Please rate the severity of the following symptoms in the last week: Tingling (pins and needles) in your arm, shoulder or hand.

- NONE
- MILD
- MODERATE
- SEVERE
- EXTREME

23. During the past week, how much difficulty have you had sleeping because of the pain in your arm, shoulder or hand?

- NO DIFFICULTY
- MILD DIFFICULTY
- MODERATE DIFFICULTY
- SEVERE DIFFICULTY
- SO MUCH DIFFICULTY THAT I CAN'T SLEEP

This Section of the survey is the Life Quality Index tool. It consists of 15 questions on IgG infusions.
Please consider your CURRENT IgG infusion process when answering these questions.

Each of the following questions should be answered by selecting a number between 7 and 1.
Both extremes (7 and 1) are described and the numbers between represent gradings between these extremes.

24. My IgG treatments...

- 1 **are inconvenient**
- 2
- 3
- 4
- 5
- 6
- 7 **are convenient**

25. My IgG treatments...

- 1 **are painful**
- 2
- 3
- 4
- 5
- 6
- 7 **are not painful at all**

26. My IgG treatments

- 1 **have not improved my health**
- 2
- 3
- 4
- 5
- 6
- 7 **have improved my health**

27. My IgG treatments...

- 1 **interfere with my social/ family life**
- 2
- 3
- 4
- 5
- 6
- 7 **do not interfere with my social/ family life**

28. My IgG treatments...

- 1 **interfere with my work/ school**
- 2
- 3
- 4
- 5
- 6
- 7 **do not interfere with my work/ school**

29. My IgG treatments...

- 1 **given in a place where I am uncomfortable**
- 2
- 3
- 4
- 5
- 6
- 7 **given in a place where I am comfortable**

30. My IgG treatments...

- 1 **require too much time waiting beforehand**
- 2
- 3
- 4
- 5
- 6
- 7 **do not require too much time waiting beforehand**

31. My IgG treatments...

- ◯ 1 **given in an unpleasant atmosphere**
- ◯ 2
- ◯ 3
- ◯ 4
- ◯ 5
- ◯ 6
- ◯ 7 **given in a pleasant atmosphere**

32. My IgG treatments...

- ◯ 1 **in my opinion, are a waste of time**
- ◯ 2
- ◯ 3
- ◯ 4
- ◯ 5
- ◯ 6
- ◯ 7 **in my opinion, are worthwhile**

33. My IgG treatments...

- ◯ 1 **make me anxious or nervous**
- ◯ 2
- ◯ 3
- ◯ 4
- ◯ 5
- ◯ 6
- ◯ 7 **do not make me anxious or nervous**

34. My IgG treatments...

- ◯ 1 **seem to me to be too expensive**
- ◯ 2
- ◯ 3
- ◯ 4
- ◯ 5
- ◯ 6
- ◯ 7 **do not seem to be too expensive**

35. My IgG treatments...

- 1 **make me too dependent on others**
- 2
- 3
- 4
- 5
- 6
- 7 **do not make me dependent on others**

36. My IgG treatments...

- 1 **require a lot of travel time and cost**
- 2
- 3
- 4
- 5
- 6
- 7 **require very little travel time and cost**

37. My IgG treatments...

- 1 **limit my freedom to take trips or move**
- 2
- 3
- 4
- 5
- 6
- 7 **do not limit my freedom to take trips or move**

38. My IgG treatments...

- 1 **not scheduled according to my convenience**
- 2
- 3
- 4
- 5
- 6
- 7 are scheduled according to my convenience

39. Considering all the different ways your disease is affecting you,
if you would stay in this state for the next few months,
do you consider that your current state is satisfactory?

- ◯ Yes
- ◯ No

40. In general, how would you rate your physical health?

- ◯ Poor
- ◯ Fair
- ◯ Good
- ◯ Very Good
- ◯ Excellent

41. To what extent are you able to carry out your everyday physical activities
such as walking, climbing stairs, carrying groceries, or moving a chair?

- ◯ Not at all
- ◯ A little
- ◯ Moderately
- ◯ Mostly
- ◯ Completely

42. In general, how would you rate your mental health,
including your mood and your ability to think?

- ◯ Poor
- ◯ Fair
- ◯ Good
- ◯ Very Good
- ◯ Excellent

43. In general, how would you rate your satisfaction with your social activities and relationships?

- Poor
- Fair
- Good
- Very Good
- Excellent

Immune Deficiency Foundation

* 44. Does any part of the treatment regimen cause you to miss work/school?

- Yes
- No

Immune Deficiency Foundation

45. How much time of work/school per month do you typically miss?

- Less than 1 hour
- Between 1-3 hours
- Half a day to one full day
- 1-2 days
- More than 2 days
- Not sure

46. How long have you been on IgG treatment?

- Less than 1 year
- 1-2 years
- 2-3 years
- 4-6 years
- 6+ years

47. How many years have you been on your current IgG treatment?

48. Which factor is MOST important to you when choosing an IVIg, SCIg or other IgG treatment?

- Time interval between dosing
- Steady-levels of treatment (IgG) in the person’s blood
- Less side effects
- Sufficient venous access
- Having a nurse infuse for the person
- The person has the ability to self-infuse (does not require a nurse to infuse
- Getting treatment in an infusion center where others are receiving infusions
- Infusing at home, no travel required
- Time taken for infusion
- Fear of inserting SC needle
- Doctor's influence/recommendation
- Other (please specify)

* 49. Has there been any change in preference for IVIg versus SCIg recently due to the COVID-19 global pandemic?

- Yes
- No
- Don't know/ not sure

50. In what direction?

- ◯ IVIg less preferred
- ◯ IVIg more preferred

51. What is the PRIMARY reason for this change in preference?

- ◯ Greater concern about infection risk during IVIg infusion visit
- ◯ Difficulties in scheduling regular infusions at infusion cente
- ◯ Other (please specify)

* 52. Did you actually switch your IgG mode of infusion due to the COVID-19 situation?

- ◯ Yes
- ◯ No

53. Did not being able to make the switch in infusion method have an impact on maintaining treatment?

- Yes
- No

54. Please select the color "Blue" from the list below.

- ◯ Yellow
- ◯ Red
- ◯ Blue
- ◯ Green
- ◯ Orange

Immune Deficiency Foundation

Intravenous immunoglobulin is infused using a single, large needle, inserted into your vein and has a large infusion bag.

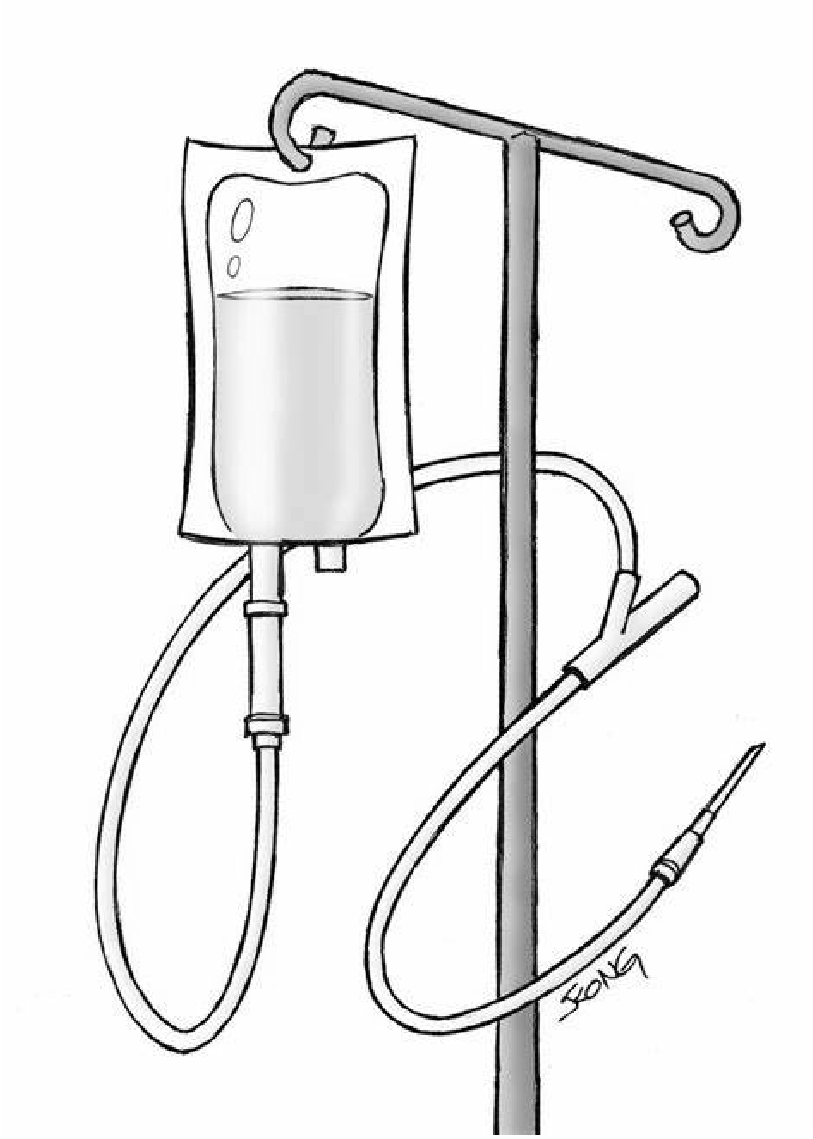

Subcutaneous immunoglobulin is typically infused with one or more small needles, inserted into your skin.

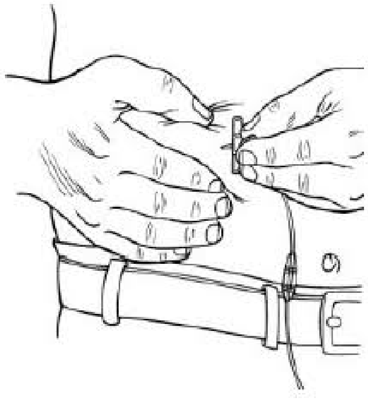

* 55. Are you currently receiving IVIg or SCIg treatment?

- IVIg
- SCIg
- Other (please specify)

56. What IVIG product do you currently use?

- ASCENIV
- Bivigam
- Flebogamma
- Gammagard Liquid
- Gammaked
- Gammaplex
- Gamunex-C
- Octagam
- Panzyga
- Privigen
- Don't know
- Other (please specify)

57. How often do you receive your IVIg treatment?

- Weekly or more frequently
- Every two weeks
- Every three weeks
- Every four weeks
- Every five weeks or more

58. How many grams of IVIg per do receive for each infusion?
Please specify in grams using a whole number, no decimals.

59. As of today, about how many weeks has it been since you received your most recent infusion?

- Less than two weeks
- 2-3 weeks
- 3-4 weeks
- More than 4 weeks

60. Where do you normally receive the infusion?

- Hospital
- Infusion center
- Home
- Other (please specify)

61. How long does it usually take for you to travel back and forth to the infusion center (Round-trip)?

Please specify in hours and minutes.

Hours

Minutes

62. How long does the pre-infusion time (including check-in, waiting, and prep) usually take?

Please specify in hours and minutes.

Hours

Minutes

63. How long does the actual infusion treatment usually take?

Please specify in hours and minutes.

Hours

Minutes

64. How long does the post-infusion time (including clean-up, waiting to check out) usually take?

Please specify in hours and minutes.

Hours

Minutes

* 65. Has your health care professional encountered difficulty finding a vein while administering IVIG?

- Yes
- No

* 66. Has it led to interruption of your IVIG infusion session?

- Yes, had to be rescheduled to different day
- Yes, for that day only
- No

67. Did the interruption require subsequent use of a central venous access device (CVAD, PORT)

- ◯ Yes
- ◯ No

Q68

68. What is your current method of SCIg administration?

- ◯ Pump with vials
- ◯ Pump with pre-filled syringe (PFS)
- ◯ Push with vials
- ◯ Push with pre-filled syringe (PFS)
- ◯ Other

69. What Scig product do you currently use?

- ◯ Cutaquig
- ◯ Cuvitru
- ◯ Gammagard Liquid
- ◯ Gammaked
- ◯ Gamunex-C
- ◯ Hizentra
- ◯ HIQVIA
- ◯ Xembify
- ◯ Don't know
- ◯ Other (please specify)

* 70. Prior to SCIg treatment, did you ever receive IVIg?

- ◯ Yes
- ◯ No

Immune Deficiency Foundation

* 71. Did you switch directly from the IVIG to your current method of SCIg administration (pump with vial, pump with PFS, push with vial, or push with PFS)?

- Yes
- No

72. What method of SCIg administration were you previously receiving?

- ◯ Pump with vials
- ◯ Pump with PFS
- ◯ Push with vials
- ◯ Push with PFS

* 73. Did you start your SCig treatment directly on your current method of SCIg administration? (pump with vial, pump with PFS, push with vial, or push with PFS)?

- ◯ Yes
- ◯ No

74. What method of SCIg administration were you previously receiving?

- ◯ Pump with vials
- ◯ Pump with PFS
- ◯ Push with vials
- ◯ Push with PFS
- ◯ Other (please specify)

Immune Deficiency Foundation

Q75

75. On average, how often do you infuse your current SCIg treatment?

- ◯ Everyday
- ◯ 2 times per week
- ◯ 3 times per week
- ◯ Weekly
- ◯ Other (please specify)
- ◯ Every 2 weeks
- ◯ Every 3 weeks
- ◯ Every 4 weeks

76. How many grams of SCIg per infusion do you receive?
Please specify in grams (g), please round-up your answer to a whole number.

* 77. How long does the preparation time before each infusion usually take for you?

Please specify in minutes.

78. How long does the actual infusion treatment usually take?

Please specify in minutes.

79. How long does the post-infusion ‘clean-up’ time usually take?

Please specify in minutes.

80. How many infusion sites do you typically use if using pump?

- ◯ 1
- ◯ 2 - 3
- ◯ 4 or more
- ◯ Does not apply, I use push method

81. How many infusion sites do you typically use if using push?

- 1
- 2 - 3
- 4 or more
- Does not apply, I use a pump

82. If you use a pump, which pump are you currently using?

- Freedom Edge (Koru)
- Freedom 60 (Koru)
- Crono Pump
- SCIg 60 Syringe Pump (EMED)
- Don't know/Not sure
- Does not apply, I use push method
- Other (please specify)

* 83. If you use a pump with Pre-Filled Syringes, does the Pre-Filled Syringe fit directly into your pump?

- Yes
- No
- Don't know/Not sure
- Does not apply, I use push method

84. If the Pre-Filled Syringe does not fit directly into your pump, do you have to do a tip-to-tip transfer of the IG from the Pre-Filled Syringe to a regular syringe that does fit into your pump?

- Yes
- No
- Does not apply, I use push method

85. If the Pre-Filled Syringe does not fit directly into your pump, is it because

- the available pump is larger than the available Pre-Filled Syringe size
- The available pump is smaller than the available Pre-Filled Syringe Size
- Does not apply, I use push method

86. If you switched from using vials to a Pre-Filled Syringe (or vice versa), how convenient was (or is) the previous method compared to the current method

- Drawing IG from vials directly into a syringe that fits the pump is more convenient than a tip-to-tip transfer of IG from a pre-filled syringe to a regular syringe that fits my pump
- Doing tip-to-tip transfer of IG from a pre-filled syringe to regular syringe that fits my pump is more convenient than drawing IG from a vial into a regular syringe
- They are both equally convenient (or inconvenient)
- Does not apply, I use push method

Immune Deficiency Foundation

Q87

This section will ask you questions about the training you received for learning methods of SCig infusion.

87. How easy or difficult was it to learn how to infuse SCIg by your current administration method?

- Very difficult
- Somewhat difficult
- Neither difficult nor easy
- Somewhat easy
- Very easy

88. How many training sessions in total did you have to learn your current method of SCIg administration?

- 1
- 2
- 3
- 4
- 5
- more than 5

89. If applicable, how many training sessions in total did you have to learn the original SCIg administration method?

- 1
- 2
- 3
- 4
- 5
- More than 5
- Not applicable, this is my original SCIg method

90. Where was the training conducted for the current method of SCIg administration?

- Hospital
- Doctor’s office
- Home
- Infusion Center/Suite
- Other (please specify)

91. If the current method of SCIg administration is different from the original method of SCIg administration; where was training conducted for the original method of SCIg administration?

- Hospital
- Doctor’s office
- Home
- Infusion Center/Suite
- Not applicable, this is my original SCIg method

92. Q92. Who conducted the training for current method of SCIG administration?

- Doctor’s office staff
- Home health / Specialty pharmacy staff
- Nurse at hospital
- Patient support program: Hizentra Nurse at home
- Patient support program: Nurse at home
- No training was provided
- Other (please specify)

93. If the current method of SCIg administration is different from the original method of SCIg administration; who conducted the training for the original method of SCIg administration?

- ◯ Doctor's office staff
- ◯ Home health / Specialty pharmacy staff
- ◯ Nurse at hospital
- ◯ Patient support program: Hizentra Nurse at home
- ◯ Patient support program: Nurse at home
- ◯ No training was provided
- ◯ Not applicable, this is my original SCIg method
- ◯ Other (please specify)

[ ]

94. On average, how long did each training session last for your current method of SCIg administration?

Please specify in hours, rounding up, or down. to the nearest hour.

[ ]

95. If applicable, on average, how long did each training session last for your original method of SCIg administration? If not applicable, please skip this question.

Please specify in hours, rounding up, or down, to the nearest hour.

[ ]

96. What were you most concerned about during training for your current method of SCIg administration?

- ◯ Drawing drug from vial into syringe
- ◯ Replacing the transfer needle/spike on syringe with the tubing
- ◯ Applying the needles to the tubing for administration
- ◯ Inserting the needle into the skin
- ◯ Priming the tubing so that it stopped short of the needle
- ◯ Attaching syringe to the pump
- ◯ Operating the pump
- ◯ Tip-to-tip syringe transfer to accommodate pump size
- ◯ I had no concerns

97. If applicable, what were you most concerned about during training for the original method of SCIg administration?

- ◯ Drawing drug from vial into syringe
- ◯ Replacing the transfer needle/spike on syringe with the tubing
- ◯ Applying the needles to the tubing for administration
- ◯ Inserting the needle into the skin
- ◯ Priming the tubing so that it stopped short of the needle
- ◯ Attaching syringe to the pump
- ◯ Operating the pump
- ◯ Tip-to-tip syringe transfer to accommodate pump size
- ◯ I had no concerns
- ◯ Not applicable, this is my original SCIg method

98. Which factors do you take into consideration when choosing a treatment? Select ONLY the TOP THREE factors taken into consideration

- ☐ Doctor recommendation
- ☐ Out of pocket cost
- ☐ Ability to reduce number and severity of infections
- ☐ Number of years the product has been available for use
- ☐ How safe the product is
- ☐ Number of people who have used the product/trusted brand
- ☐ Support programs available by maker of treatment
- ☐ Other (please specify)
- ☐ Multiple vial or PFS sizes to accommodate your needs
- ☐ Side effects
- ☐ Insurance will cover treatment
- ☐ Ease of self-administration
- ☐ Availability of PFS
- ☐ Time it takes to administer

* 99. Are you currently using a different method of SCIg administration that you have in the past?

- ◯ Yes
- ◯ No

100. If you switched from a previous method of SCIg administration to the current method, why was this switch made? Please select the TOP reason

- Problems with drawing solution up from vial
- Problems with tip-to-tip syringe transfer
- Needle phobia
- Prefer to keep hands free during infusions
- Better control over infusion speed
- Doctor recommendation
- Friend recommendation
- Hand weakness
- Easier/simpler self-administration method
- Other (please specify)

101. How did switching from the previous SCIg administration method to your current SCIg administration method impact your satisfaction with treatment?

- Substantially worsened treatment satisfaction
- Worsened treatment satisfaction
- Treatment satisfaction remained the same
- Improved treatment satisfaction
- Substantially improved treatment satisfaction

102. How did switching from the previous SCIg adminstration to your SCIg administration method impact your productivity (including time lost) at usual activities (work, school, home or other)?

- Substantially worsened productivity at usual activities
- Worsened productivity at usual activities
- Productivity at usual activities remained the same
- Improved productivity at usual activities
- Substantially improved productivity at usual activities

103. How did switching from the previous SCIg administration to your current SCIg administration method impact your compliance to labeled frequency and dose of therapy?

- Substantially worsened compliance to labeled frequency and dose of therapy
- Worsened compliance to labeled frequency and dose of therapy
- Compliance to labeled frequency and dose of therapy remained the same
- Improved compliance to labeled frequency and dose of therapy
- Substantially improved compliance to labeled frequency and dose of therapy

104. Q104. How important to you is the time taken per infusion?

- Completely unimportant
- Mostly unimportant
- Neither important nor unimportant
- Somewhat important
- Very important

DEMOGRAPHICS

105. Do you currently have any permanent impairment or loss of…?

Please select all that apply.

- ☐ Digestion
- ☐ Hearing
- ☐ Kidney function
- ☐ Liver function
- ☐ Lung function
- ☐ Mobility
- ☐ Hand/ eye coordination
- ☐ Neurological function
- ☐ Vision
- ☐ No permanent loses
- ☐ Other (please specify)

106. What kind of health insurance do you currently have? (select all that apply)

- ☐ Employer sponsored group plan
- ☐ COBRA
- ☐ Individual policy (for individuals or family)
- ☐ Medicare A & B
- ☐ Medicare Supplemental Plan
- ☐ Medicare Advantage Plan
- ☐ Medicare due to disability
- ☐ Medicaid
- ☐ Other government policy
- ☐ State Excange or Markeplace
- ☐ Federal Excahnge or Markeplace
- ☐ TRICARE
- ☐ Veterans Policy
- ☐ Other Insurance
- ☐ No health insurance

107. Which of the following categories would best describe your race or ethnicity?

- American Indian/Alaskan native
- Asian/Pacific Islander
- Black/African-American
- Hispanic or Latino/a
- White, non-Hispanic
- Two or more races
- Other
- Prefer not to answer

108. What is your current employment status?

- Employed full-time
- Employed part-time
- Unemployed, looking for work
- Student
- Homeaker
- Disabled/ too ill to work
- Other
- Prefer not to answer

109. What is the last grade or year of school you completed?

- 8th grade or less
- Some high school
- High School Graduate/GED
- 1-3 years of college
- 4- year college graduate
- Graduate Degree
- Prefer not to answer

110. In which U.S. state do you currently reside?

111. What was your household’s total income last year?

- Under $15,000
- Between $15,000 and $29,999
- Between $30,000 and $49,999
- Between $50,000 and $74,999
- Between $75,000 and $99,999
- Between $100,000 and $150,000
- Over $150,000
- Prefer not to answer

That completes the survey, thank you for your participation and time!

112. If you would like to be entered into a raffle for a chance to win one of the three Amazon Coupon Code prizes, please enter your e-mail address below.

Email Address

If you have any questions or concerns about this survey,
please e-mail us at idfresearch@primaryimmune.org

We are sorry you do not qualify for this survey.

Thank you for your time and your interest!

If you have any questions or concerns about this survey, please e-mail us at idfresearch@primaryimmune.org
